# Influence of the Mold Material on the Injection Molding Cycle Time and Warpage Depending on the Polymer Processed

**DOI:** 10.3390/polym13183196

**Published:** 2021-09-21

**Authors:** Thomas Lucyshyn, Lara-Vanessa Des Enffans d’Avernas, Clemens Holzer

**Affiliations:** Department of Polymer Engineering and Science, Polymer Processing, Montanuniversitaet Leoben, Otto Gloeckel-Strasse 2, 8700 Leoben, Austria; lara-vanessa.davernas@stud.unileoben.ac.at (L.-V.D.E.d.); Clemens.Holzer@unileoben.ac.at (C.H.)

**Keywords:** injection molding, simulation, thermal conductivity, cycle time, Moldflow, warpage, thermoplastics, mold material

## Abstract

The thermal properties of the mold influence the cooling situation in the injection molding process. While there are experimental studies investigating the influence of special mold materials, they are limited to few polymers. In this work, an extensive parameter study with the simulation software Autodesk Moldflow Insight was performed to analyze the influence of the polymer itself on the impact of the mold steel on cycle time and warpage. The investigated part was a box with two thickness variations. A conventional mold steel was compared with a steel grade featuring approximately double the thermal conductivity. Simulations were performed with 18 polymers covering the most common material families. The main finding of this study was that the influence of the higher mold conductivity on cycle time ranged from an almost negligible reduction (3%) up to a strong effect (24%), depending mainly on the used polymers, but also on the part thickness. For the cycle time reduction, a correlation was found, with the melt, mold and ejection temperature being the dominant influencing factors of the polymers. With this correlation, it was possible to estimate the potential of cycle time reduction for other polymers. The simulations also showed a positive influence of the higher mold thermal conductivity on part warpage.

## 1. Introduction

Cycle time is a crucial economic factor of the injection molding process. Several factors potentially affect the cycle time, such as the thermal properties (thermal conductivity and specific heat capacity) of the polymer and the mold material, the geometry of the injection molded part (specifically the thickness), and the layout of the cooling channels or the chosen process settings (especially the melt and mold temperatures). A theoretical description of the dependence of the cooling time on many of these parameters has already been published about 40 years ago by Zorić and Čatić [1,2]. With a defined polymer and part geometry, the mold material is one potential degree of freedom to reduce cycle time. According to Čatić et al. [3] the thermal properties of the mold affect the cycle time in two ways: the thermal conductivity of the mold material influences the contact temperature at the metal/polymer interface, and conversely, the heat transport from the interface into the mold is supported by a higher thermal conductivity of the mold material.

Experimental work with focus on the influence of the thermal properties of the mold material on cycle time, part quality, and energy consumption was presented by Kelly et al. [4]. In their study, the authors compared a conventional tool steel with a thermal conductivity of 20 W·(m·K)^−1^ to beryllium-free copper alloys with rather high copper contents from 85% to 96% and thermal conductivities up to 208 W·(m·K)^−1^. The investigations were performed for a simple plate shaped part with two semi-crystalline materials (high density polyethylene and poly butyl terephthalate). In this publication, already a difference of the effectivity of the mold material was stated between the two polymers but not investigated in more detail. This study also showed the difficulties in the experimental determination of the molded part temperature.

Other relevant literature also focuses mainly on the influence of different mold materials, cooling channel design or process settings on mechanical part properties [5] or cycle time and warpage [6,7,8,9,10], without investigating if the influence of the mold material varies with different polymers.

In the presented work, a parameter study using the commercial injection molding simulation software Autodesk Moldflow Insight 2016 (AMI) was performed to better understand the influence of the polymer itself on the effectivity of the mold material in terms of cycle time reduction. Due to the aforementioned difficulties in temperature measurement described by Kelly et al. [4] and based on the positive evaluation of the cooling simulation accuracy with the software AMI [6], a simulation approach was chosen but was still additionally attempted to validate experimentally using an IR-camera. In order to obtain a representative overview on industrially relevant materials, 18 different polymer types were investigated covering the most common polymer families also considering different morphologies (amorphous vs. semi-crystalline or unfilled vs. fiber reinforced). As a side topic, apart from the cycle time, the influence of the mold material on warpage, filling pressure and clamp force was also investigated for the 18 polymers.

## 2. Materials and Methods

### 2.1. Simulation

**Figure 1:**
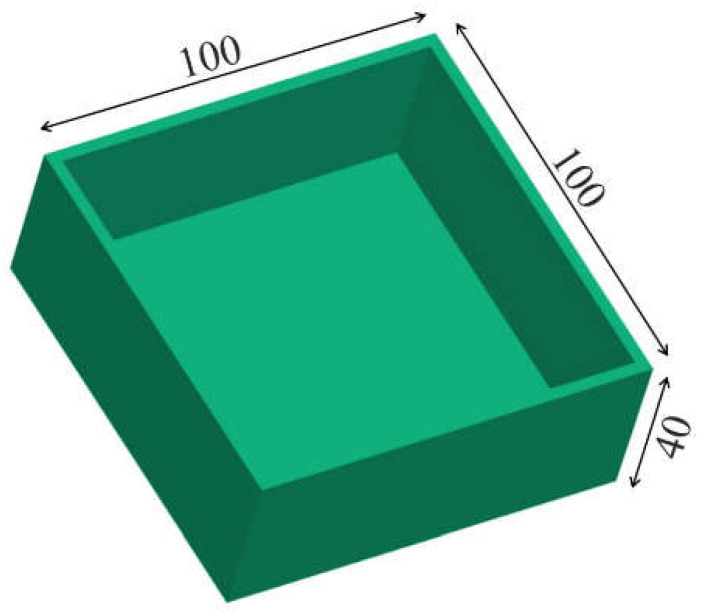
Geometry of the investigated box shaped part with global dimensions in mm.

For the simulations, the commercial injection molding software Autodesk Moldflow Insight 2016 (Autodesk, San Rafael, CA, USA) was used. The investigated test part had a box shaped geometry with a bottom area of 100 by 100 mm^2^ and a height of 40 mm (Figure 1). The thickness was varied with either 1 or 3 mm for the whole part to see the thickness influence on the results.

**Figure 2:**
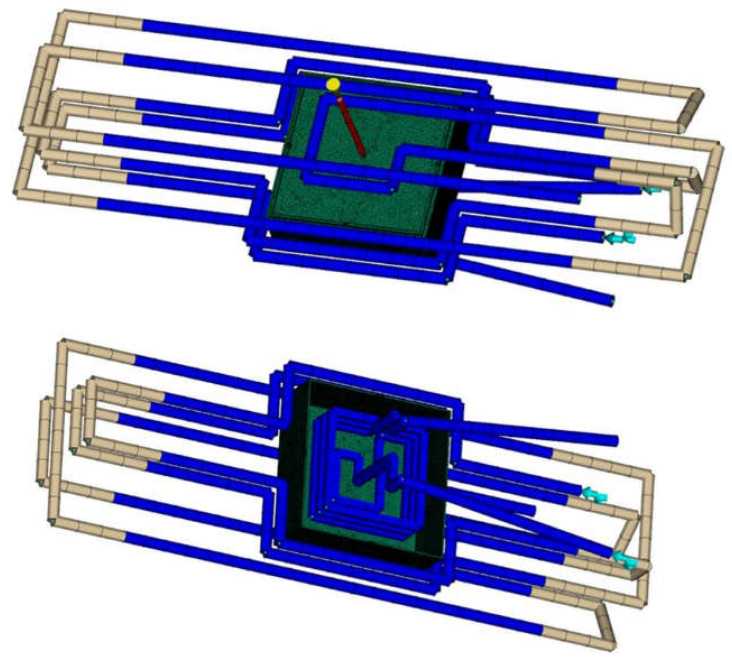
Simulation model with part (green), cooling system (blue/gray) and hot runner (red).

The gating situation was a hot runner with a short cold gate to the center of the part bottom. The short cold gate was modeled in order to avoid a hot spot on the part at the contact node with the hot runner, which is always kept at melt temperature in the simulation model. The part was meshed with a 3D tetrahedral mesh with 8 elements over the thickness (~740,000 elements) and an average surface edge length of 1.5 mm. In order to better capture the high temperature gradients toward the surface of the part, the biasing option in Moldflow was used producing a coarser mesh in the core and a finer mesh toward the surface of the part. The hot and cold runner as well as the cooling channels were modeled with beam elements. The whole mold block (with cross sectional dimensions perpendicular to the opening direction of 295 × 295 mm^2^ and a mold height of 350 mm) was meshed with a 3D tetrahedral mesh (~2.6 m. elements). The part with the cooling system and hot runner is shown in Figure 2.

The whole mold was defined either as a commercial tool steel 1.2343 ESU or a special test steel alloy with higher thermal conductivity, further referred to as “test alloy” (summary of thermodynamic properties in Table 1), both from voestalpine BÖHLER Edelstahl GmbH & Co KG (Kapfenberg, Austria). The main difference between the two steel grades is their thermal conductivity with 26 W·(m·K)^−1^ for 1.2343 ESU and 49 W·(m·K)^−1^ for the test alloy.

In order to analyze the influence of the thermal conductivity of the mold material on the cycle time for different polymers, 18 widely used material families were arbitrarily chosen from the Moldflow database and the recommended processing temperatures (melt, mold, and ejection temperature) were used for the simulations (Table 2). For the selection of materials, a mixture of amorphous and semi-crystalline materials as well as unfilled and glass fiber reinforced materials was chosen to cover a representative range of industrially relevant polymer grades. A transient 3D-FEM cooling analysis was performed, assuming a stable production process (“transient within cycle” option in Moldflow), followed by a filling, packing and warpage simulation.

The most relevant governing equation for the simulation of the mold and part temperature is the energy conservation equation [11], which is used in its general form for the polymer (during filling, packing and cooling) in Cartesian coordinates according to Equation (1):

(1) $${\rho c}_{p}\left(\frac{\partial T}{\partial t}+v_{x}\frac{\partial T}{\partial x}+v_{y}\frac{\partial T}{\partial y}+v_{z}\frac{\partial T}{\partial z}\right)=\beta T\left(\frac{\partial p}{\partial t}+v_{x}\frac{\partial p}{\partial x}+v_{y}\frac{\partial p}{\partial y}+v_{z}\frac{\partial p}{\partial z}\right)+{\eta \dot{\gamma }}^{2}+k\left(\frac{\partial^{2}T}{\partial x^{2}}+\frac{\partial^{2}T}{\partial y^{2}}+\frac{\partial^{2}T}{\partial y^{2}}\right),$$

where ρ is the density, c_p_ is the specific heat capacity, T is the temperature, t is the time, v_i_ are the velocity components, x, y, and z are the coordinate directions, β is the compressibility, p is the pressure, η is the shear viscosity, $\dot{\gamma }$ is the shear rate, and k is the thermal conductivity with all parameters being material properties of the polymer.

The heat transfer within the mold is described with a simplified energy conservation equation, which is basically a transient 3D heat conduction problem according to Equation (2) with the material parameters of the mold material, which is solved by FEM:

(2) $${\rho c}_{p}\frac{\partial T}{\partial t}=k\left(\frac{\partial^{2}T}{\partial x^{2}}+\frac{\partial^{2}T}{\partial y^{2}}+\frac{\partial^{2}T}{\partial z^{2}}\right)$$

For the heat transfer coefficient (HTC), the default values of Moldflow were used (5000 W·(m^2^K)^−1^ during filling, 2500 W·(m^2^K)^−1^ during packing and 1250 W·(m^2^K)^−1^ when detached). Due to a lack of measured individual HTC values for each polymer, the influence of this factor was evaluated by existing literature, and conversely, by an exemplary parameter study. Heinle and Drummer published a review paper on the topic of the HTC in injection molding [12] covering reported measurement methods as well as HTC values depending on different influencing parameters. The reported values are in the range of 500 up to 14,000 W·(m^2^K)^−1^, which shows the difficulty in reliably measuring/determining the heat transfer coefficient, but also that it is influenced by a variety of parameters such as pressure/contact conditions, surface roughness, etc. The effect of the HTC is specifically relevant for either thin-walled parts (<1 mm) [13] or microstructured regions on macroscopic parts. Nguyen-Chang et al. [14] investigated the influence of the HTC value for microinjection molding with part thicknesses of 0.2 and 0.5 mm, which is significantly lower than the values used in our experiments. For these thin walled parts, the HTC value had a significant influence on the simulation results. Contrary to that, other investigations published by Nylund and Meinander [15] showed that for parts in the thickness range from 2 to 5 mm, the temperature of the centerline of the part was not significantly influenced by the HTC value, if it was above approx. 2000 W·(m^2^K)^−1^. This means that the default values of Moldflow during filling and packing are well above this value, and therefore, any deviations of the HTC from these default values within a realistic range will not significantly affect the cooling time results. In the exemplary parameter study, simulations were performed with the square box and an ABS/PC grade using the default values for HTC of Moldflow, and additionally, simulations with HTC values were 20% higher and 20% lower than the default values. With these values, the cycle times (based on the time to reach ejection temperature in the hot spot of the part) were shifted by about 2% for the 3 mm box and about 3% for the 1 mm box (as expected there was more influence on the thinner part). This influence is by far smaller than the found main influence of the processing temperatures in this study, which will be described in more detail in Section 3, Results and Discussion. Therefore, the HTC value was considered to have only a minor influence on the main conclusions of this study.

**Figure 3:**
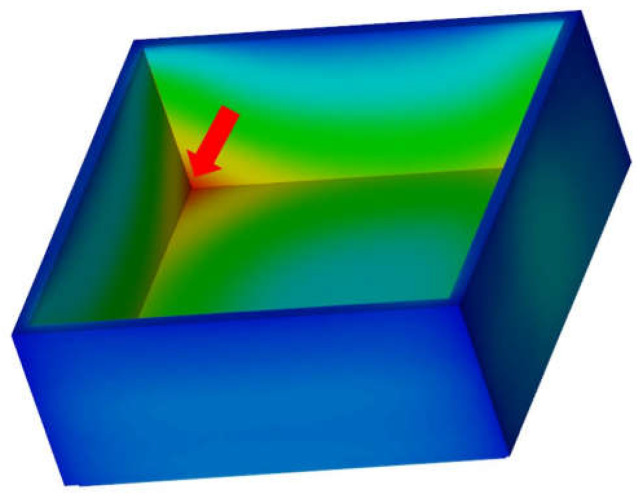
Hot spot in the corner of the part.

For both wall thicknesses (1 and 3 mm) and each of the polymers, an individual optimal packing and rest cooling time was determined for the 1.2343 ESU steel grade with several iterative simulation loops. The found parameters for each polymer were then also used for the test alloy. For both wall thickness variations, the hotspot of the part was identified in the corners of the box, close to the part surface being in contact with the mold core, where the heat coming from the polymer cannot be carried away quickly enough into the mold core (region indicated with the red arrow in Figure 3). This region was determining the required cycle time. Therefore, a specific node in one of these corners was selected for both wall thickness variations for the evaluation of all polymers to obtain comparable results. For the comparison of the cycle times, the part temperature result of this node for different process time steps was exported from Moldflow. These discrete temperature/time results were then approximated with a polynomial function. With this function, the exact time could be calculated, at which the recommended ejection temperature for the different materials was reached at the hot spot of the part. Eventually, this time was defined as the shortest possible time for ejection. Adding a mold open time of assumed 5 s (for opening and closing the mold as well as ejecting the part) resulted in the evaluated cycle time for each individual polymer, mold material, and wall thickness. The cycle time reduction of the test alloy was expressed in percent relative to the reference cycle time of the 1.2343 ESU steel grade.

Although the main focus of this study was on the cycle time, additionally, the distance of opposite side walls on the open side of the box was evaluated in terms of shrinkage and warpage to analyze the influence of the tool steel on this quality criterion as well. For that purpose, a pair of nodes on the center of the opposite side walls was defined for each wall thickness variation. The distance of these nodes before and after deformation was evaluated for each individual polymer, mold material and wall thickness variation.

Furthermore, the injection pressures and resulting clamp forces were also analyzed in order to see a potential negative influence of the higher thermal conductivity of the mold steel on the processability of the polymers.

### 2.2. Experimental Validation of Cycle Time Reduction

**Figure 4:**
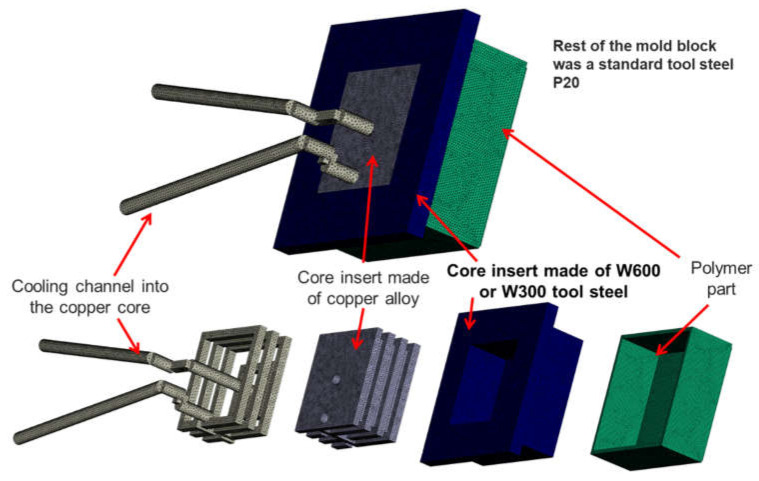
Mold configuration for the validation experiments.

In order to validate the simulation results regarding the cooling time reduction, experiments were performed with two selected materials—a polypropylene (PP) and a polycarbonate (PC) grade. In order to reduce the infrared (IR) transparency of the two polymers, they were colored with dark color masterbatches. For the validation, only the core insert was composed of the two different mold materials W300 and W600 from voestalpine BÖHLER Edelstahl GmbH & Co KG (Kapfenberg, Austria). The concrete configuration of the mold with different materials is shown in Figure 4, and the corresponding thermodynamic material properties of the used mold materials are listed in Table 3.

**Figure 5:**
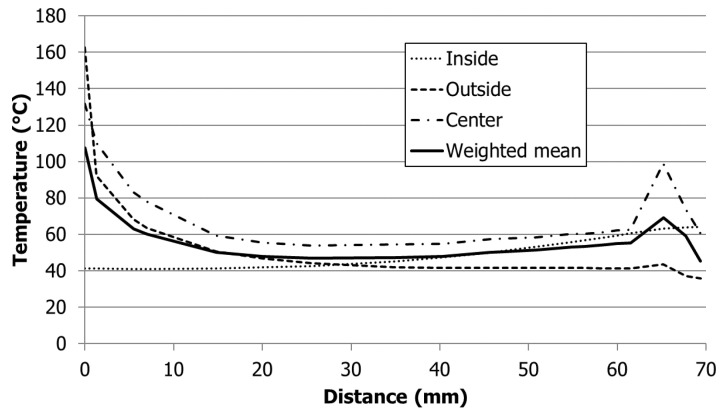
Simulated temperatures for a PP for the 1 mm box on a diagonal line from the center of the part to one corner at the inside surface, center and outside surface of the box and weighted mean temperature along the diagonal.

An IR camera (FLIR SC7000 from FLIR Systems Inc., Wilsonville, OR, USA) was first calibrated by using boxes of the two colored materials floating on temperature controlled water and measuring the infrared signal together with a conventional thermometer in the water bath. This measurement was repeated at different water temperatures. The emissivity setting of the IR camera was then adjusted to obtain the same temperature readings as on the thermometer. Despite the coloring of the materials and being optically opaque, the materials still turned out to be partly transparent to IR radiation to a certain extent, which caused major difficulties in the evaluation of the temperature signal when measuring the part in production. The camera was positioned on top of the moving mold half, and a robot took the part from the mold at the end of the cycle and positioned it in front of the camera for temperature measurement of the inside of the box. To compare the experimental data with the simulation data, a diagonal line from the center of the part to one of the corners (with the hot spot) was defined and evaluated in the IR camera results as well as the simulation results. Due to the IR transparency of the materials, the camera measured the radiation energy from the volume of the part with a higher intensity coming from the surface pointing toward the camera and a lower intensity from the opposite part surface. Contrary to that, in the simulation, the results were only available at certain discrete points. In order to be able to compare the simulation results with the experimental results, three evaluations along the defined diagonal were performed, one on the inside surface, one in the center, and one on the outside surface of the box. These three temperatures over the thickness were then weighted with higher weights on the inside, which is pointing toward the camera (Figure 5). For the given example of the 1 mm thick box of PP, the weights were 0.37 for the inside, 0.33 for the center and 0.3 for the outside. This was the best possible way to be able to compare the local temperature results from the simulation with the volumetrically based temperature results from the IR camera.

## 3. Results and Discussion

### 3.1. Cycle Time Reduction

**Figure 6:**
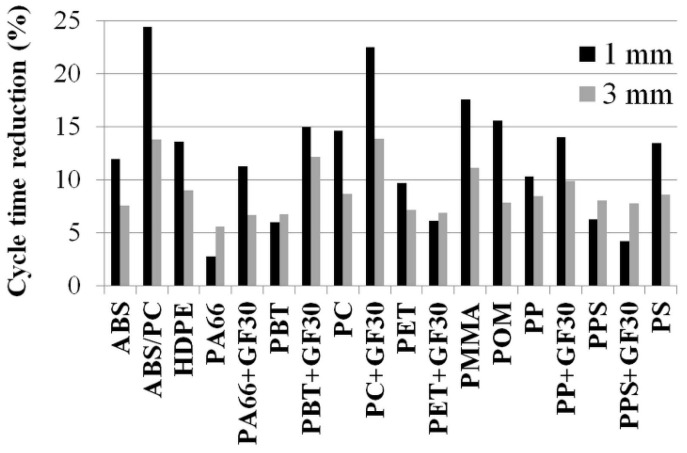
Cycle time reduction of test alloy compared to 1.2343 ESU for different polymers and two wall thicknesses.

As expected, the steel with the higher thermal conductivity resulted in a reduction of cycle time. What was surprising was the range of values from about 3% to 24% of cycle time reduction for the 1 mm box and approximately 6% to 14% for the 3 mm box (Figure 6 and Table A1 and Table A2).

The influence of the wall thickness can be explained by the low thermal conductivity of the polymer itself. Generally spoken, with increasing part thickness the effect of the surrounding steel becomes less important, as the heat has to be transferred from the center of the polymer part to the surface, which is mainly determined by the (poor) thermal conductivity of the polymer.

In order to find out which parameters influence the effect of the mold steel most, an additional design of experiments (DoE) with a “model material” with systematically varied material properties (thermal conductivity, specific heat capacity) and process parameters (melt temperature T_melt_, mold temperature T_mold_ and ejection temperature T_ejection_) of the polymers was performed. However, using this artificial “model material” did not work as expected because a long cycle time had to be defined to ensure that, for all the settings of the DoE, the recommended ejection temperature would be reached. Therefore, the cycle time was considerably longer than necessary for many settings, which resulted in weakening the effect of the mold steel (if a long cooling time is chosen, the core will not heat up from cycle to cycle in the same way as in the case of an optimized short cooling time, and therefore, the steel thermal properties do not have a strong impact anymore). Nevertheless, this DoE gave an indication that the three mentioned temperatures would be the most important factors influencing the effect of the mold steel on cycle time reduction. Therefore, a correlation was searched between the achieved cycle time reduction and a function incorporating these three temperatures. As a function describing the cooling time depending on those three temperatures, well known as “cooling time equation” [1], already exists, it was used as a basis and is shown in Equation (3) for a plate-shaped geometry and related to the temperature in the center of the part:

(3) $$t_{c}=\frac{s^{2}}{\pi^{2}\times a_{\mathrm{eff}}}\times \ln\left(\frac{4}{\pi }\times \frac{T_{\mathrm{melt}}-T_{\mathrm{mold}}}{T_{\mathrm{ejection}}-T_{\mathrm{mold}}}\right),$$

t_c_ is the cooling time, s is the local part thickness and a_eff_ is the effective thermal diffusivity, which is the ratio of the averaged values of the thermal conductivity divided by the product of density and specific heat capacity.

A similar relation was established defining the cycle time reduction (CTR expressed in %) as a function of a temperature coefficient (TC) containing these temperatures according to the following Equation (4):

(4) $$\mathrm{CTR}=f\left(\mathrm{TC}\right)=f\left(\frac{T_{\mathrm{melt}}-T_{\mathrm{mold}}}{T_{\mathrm{ejection}}-T_{\mathrm{mold}}}\right)$$

After testing different functions, a simple linear relation (Equation (5)) between the temperature coefficient defined in Equation (4) and the corresponding cooling time reduction with two coefficients a and b turned out to produce the best correlation coefficient R^2^:

(5) $$\mathrm{CTR}=a\times \frac{T_{\mathrm{melt}}-T_{\mathrm{mold}}}{T_{\mathrm{ejection}}-T_{\mathrm{mold}}}+b$$

**Figure 7:**
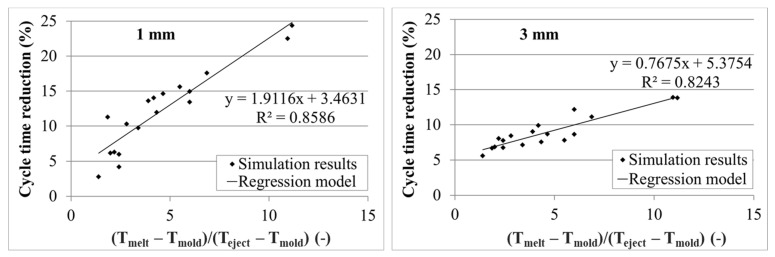
Correlation between temperature coefficient (TC) and cycle time reduction (CTR) for two wall thicknesses (left: 1 mm, right: 3 mm).

As the cooling behavior was different for the two wall thicknesses, individual regression models for both thickness variations were established. In Figure 7, the cooling time reductions for 1 and 3 mm wall thickness as a function of the temperature coefficient TC are shown together with the found correlation functions and the corresponding correlation coefficients. With an increasing value of the temperature coefficient, more cycle time reduction can be achieved. For the thicker part, a smaller slope of the function was determined.

**Figure 8:**
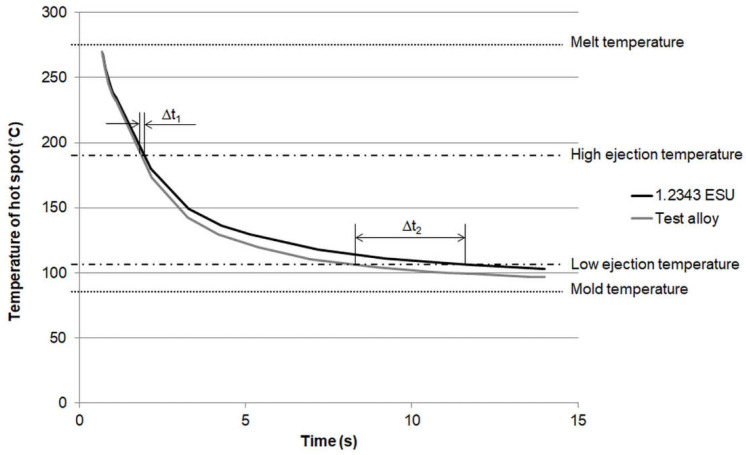
Temperature of the hot spot of the box as a function of time for the ABS/PC grade and a wall thickness of 1 mm for both investigated mold materials.

This found correlation is further explained with Figure 8, where the temperature of the defined hotspot as a function of time is shown for the ABS/PC grade and the box with 1 mm wall thickness. Additionally, the melt and mold temperatures as well as two cases of ejection temperatures (high and low) are shown in this graph. The denominator of the temperature coefficient (T_ejection_ − T_mold_) defines the slope of the cooling curve at the ejection temperature. In case of a significant difference between the ejection temperature and the mold temperature (high ejection temperature in Figure 8), the temperature gradient ΔT/Δt_1_ is high at the ejection temperature, which means that the occurring temperature difference ΔT due to the different thermal mold properties results only in a small time difference Δt_1_. In case of a small difference between the ejection and mold temperature (low ejection temperature in Figure 8), the temperature gradient becomes significantly smaller at the ejection temperature, and therefore, a similar temperature difference ΔT to the previous case results in a considerably higher time difference Δt_2_. The numerator of the temperature coefficient (T_melt_ − T_mold_) influences the temperature gradient especially at the beginning of the cooling process.

Apart from this main effect, an additional influence comes from the fact that, for a part producer, only the total cycle time (reduction) is relevant. The total cycle time consists of the three main parts injection time, cooling time (packing + residual cooling time) and mold open time (mold opening, demolding and mold closing). The thermal mold properties only affect the cooling time and have no significant effect on the injection and mold open time. In case of a short cooling time, the proportion of this time in relation to the total cycle time becomes relatively small compared to a longer total cycle time. Therefore, at shorter total cycle times, the effect of the cooling time reduction is diminished. If for example the injection time is 1 s, then the cooling time is 30 s and the mold open time is 5 s, a relative cooling time reduction of 10% (3 s) resulting in a total cycle time reduction of 1 − (33 s/36 s) = 8.3%. If the cooling time is only 10 s and the other two times (1 s injection and 5 s mold open time) remain unchanged, the same relative cooling time reduction of 10% (1 s) would result in a total cycle time reduction of only 1 − (15 s/16 s) = 6.3%. While for most of the investigated materials the cycle time reduction for the 1 mm box was higher than for the 3 mm box, for certain materials, it was not (PA66, PBT, PET+GF30, PPS, PPS+GF30). What those materials have in common is an extremely short cooling time for the 1 mm box. In these cases, the mold open time (5 s) becomes the most dominant component of the total cycle time, and therefore, the impact of the cooling time on the total cycle time is small. For the 3 mm box, the cooling time is significantly longer compared to the 1 mm box, and therefore, the cooling time becomes the dominant component of the total cycle time again. Thus, the cooling time reduction for the 3 mm box has more impact on the total cycle time reduction than for the 1 mm box, although the relative cooling time reduction itself is larger for the 1 mm box.

The correlation coefficient R^2^ for both thickness variations is not perfect, but is satisfying with values higher than 0.8. For a better correlation, the thermal diffusivity of the polymer is considered to have an influence, but as the individual thermodynamic properties, which define the thermal diffusivity (thermal conductivity, density and specific heat capacity) are all depending on temperature and the density is additionally depending on pressure, it is hard to define a single value for the thermal diffusivity that could be used in the found correlation equation. Thus far, no reasonable general model incorporating these properties was found that further increased the correlation coefficient.

**Figure 9:**
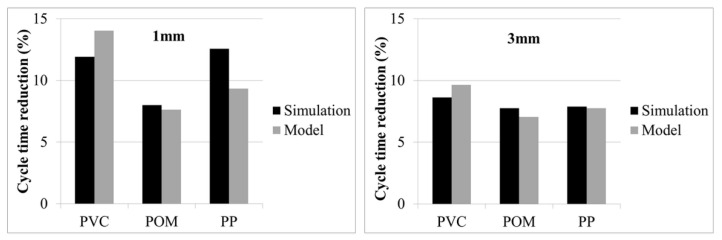
Validation of correlation model for three randomly chosen polymers for two wall thicknesses (left: 1 mm, right: 3 mm).

In order to validate the found correlation models, three additional polymers were randomly chosen from the Moldflow database (Table 4). For these polymers, the same simulations were performed and evaluated as already described for the other 18 investigated polymers for both wall thicknesses. Additionally, the expected cycle time reduction was calculated using the three given process temperatures from the Moldflow database and the correlation functions shown in Figure 7. The results of the simulations and the correlation model are compared for 1 and 3 mm wall thickness in Figure 9 and Table A3. The model is able to predict the expected cycle time reduction in a satisfying quality, although there are quantitative differences most likely explained by the different thermodynamic properties of the polymers, which were not considered in the model.

### 3.2. Experimental Validation of Cycle Time Reduction

**Figure 10:**
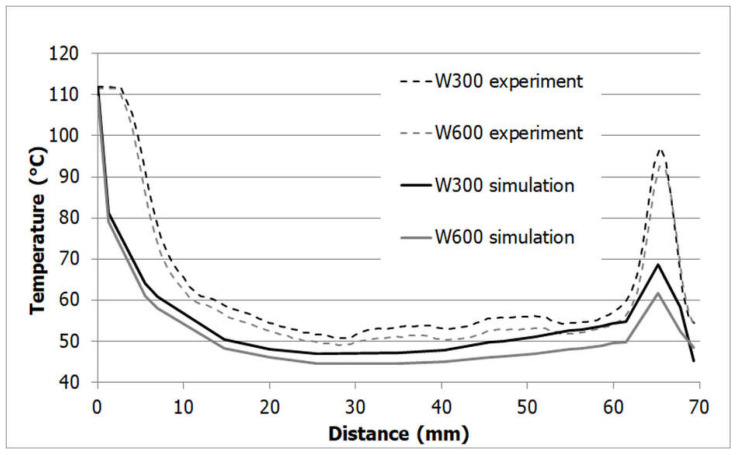
Comparison of part temperature from experiment and simulation for a PP for a 1 mm box on a diagonal line from the center of the part to one corner.

Due to the experimental limitations of the IR measurements and the different evaluation methods between experiment and simulation, a reliable quantitative comparison was not possible. The trends of the temperatures were comparable, however, between the simulation and experimental results. In Figure 10 an example of the results for the 1 mm box and PP is shown along a diagonal line from the center of the part (position 0 mm) to one corner of the part. The dotted lines represent the simulation results and the full lines the experimental data. The black lines correspond to the results obtained with the W300 insert, and the gray lines represent the results obtained with the W600 insert. The high temperatures in the center of the part result from the hot runner, which is permanently at melt temperature, and therefore, the part stays hot at this position. The temperature peaks at the corner of the part are a result of the overheating of the mold insert corners as the heat from the polymer is transferred into the mold from three sides, but it can only be carried away in one direction. This elevated temperature at the mold core results in a higher part temperature. This is also the position where the higher mold thermal conductivity has its best effect. While the absolute values do not match due to the limitations in the experiment as well as the evaluation of the simulation described in Section 2.2, the global trends are similar, and especially, the temperature differences between the two mold inserts are similar for simulation and experiment, which is the most important value, as it is used for the evaluation of the cycle time reduction. Based on these results we conclude that the simulation is sufficiently reliable to determine the temperature difference and hence the relative cycle time reduction for the different mold materials.

### 3.3. Warpage, Filling Pressure and Clamp Force

**Figure 11:**
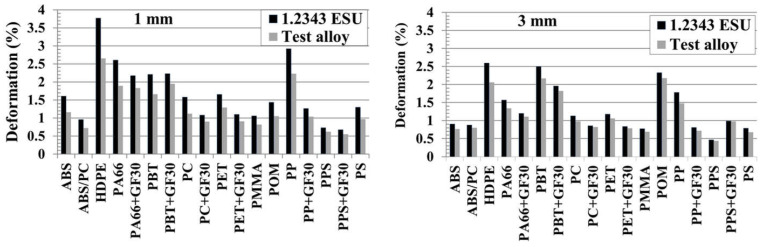
Part warpage (deformation) for test alloy compared to 1.2343 ESU for different polymers and two wall thicknesses (left: 1 mm, right: 3 mm).

Besides the focus on the cycle time reduction potential of the steel with the higher thermal conductivity, there was also a positive effect expected on the warpage behavior, especially for this box shaped part, where the core tends to overheat. The results shown in Figure 11 and Table A4 confirmed this expectation. For each material and both wall thickness variations, the warpage (bending inwards of the sidewalls) was reduced when using the test alloy. The effect was stronger for the thin walled part, with a warpage reduction of up to 30% compared to a maximum of 20% of warpage reduction for the thick walled part. The well-known positive effect of the higher mold thermal conductivity, which is based on the better temperature homogeneity of the mold between the core side and the cavity side of the mold, was confirmed with this study. With a lower thermal conductivity of the mold, the core tends to heat up, causing a higher temperature of the part at the inside of the box, resulting in the inward bending of the side wall (well-known corner effect). With a higher thermal conductivity of the mold, the core became cooler, and therefore, the temperature difference between the inside and outside became smaller, resulting in less inward bending. Contrary to the cycle time reduction, there was no strong correlation expected and found with the temperature coefficient TC.

Additionally, the effect of the higher thermal conductivity of the mold steel on the filling pressure and clamp force was evaluated. Potentially, there could have been a negative effect of the higher cooling efficiency of the steel on the filling behavior, as an improved cooling of the melt during the filling stage could result in higher viscosities and a thicker frozen layer. The simulation results proved that this effect can be neglected, as the highest increase of filling pressure as well as clamp force of all variations was smaller than 5%, which is not problematic for the processability of the parts.

## 4. Conclusions

In an extensive parameter study using the commercial injection molding simulation software Autodesk Moldflow Insight 2016, the influence of the thermal properties of the mold steel on the cycle time, the warpage, filling pressure, and clamp force were investigated for 18 widely used polymer families. The main finding was that the effect of a higher thermal conductivity of the mold steel on the cycle time (“cycle time reduction”) varied strongly among the different polymers (in the range of 3% to 24%), but also, an influence of the wall thickness was shown. As the most important influencing factor of the polymer, a temperature coefficient was identified, comprising the recommended processing temperatures (melt, mold and ejection). A linear function could be found as correlation model between this temperature coefficient and the cycle time reduction, which was validated using three additional randomly chosen polymers. With this model, it was possible to estimate the achievable cycle time reduction without having to perform several simulations, but only by using these three process parameters from the Moldflow database in the found model. Due to experimental limitations, a quantitative validation of the temperatures turned out to be impossible, but the relative differences obtained in experiment and simulation turned out to be sufficiently accurate, but not compromising the main findings of the simulation regarding the cycle time reduction. 

The results showed a positive effect of the higher thermal conductivity of the mold steel on the warpage behavior, especially for the inward bending of the side walls, which is a result of the overheating of the core. The effect of the steel on warpage reduction did not considerably depend on the used polymer. Furthermore, no negative effects on the processability in terms of filling pressure and clamp force were detected.

As a final conclusion, the results showed that the potential of cycle time reduction by using a tool material with significantly higher thermal conductivity is strongly dependent on the used polymer, but also on the part thickness, and can range from hardly any effect to a strong reduction of cycle time.

## Figures and Tables

**Table 1:** Thermodynamic properties of the two investigated mold steels.

Mold Property	1.2343 ESU	Test Alloy
Thermal conductivity (W m^−1^ K^−1^)	26	49
Specific heat capacity (J kg^−1^ K^−1^)	480	473
Density (kg m^−3^)	7780	7944

**Table 2:** Investigated polymers with filler content (GF30 = 30 wt.% glass fiber) and recommended processing temperatures (according to Moldflow material data base in the software version 2016).

Polymer	Filler	Grade Name	Manufacturer	Melt Temp. (°C)	Mold Temp. (°C)	Ejection Temp. (°C)
ABS	-	Lustran ABS LGA	INEOS ABS	226	44	86
ABS/PC	-	Cycoloy LG8002	SABIC Innov. Pl. US, LLC	277	87	104
PE-HD	-	Marlex 9004 PE	Chevron Philips	234	27	80
PA 6.6	-	Zytel ST801HS NC010	DuPont Perf. Polymers	280	70	220
PA 6.6	GF30	Ultramid A3WG6	BASF	290	85	195
PBT	-	Pocan B 1505	Lanxess	270	100	170
PBT	GF30	Ultradur B 4300 G6 HR	BASF	260	80	110
PC	-	Lexan ML7681	SABIC Innov. Pl. US, LLC	285	85	128
PC	GF30	Lexan 3413	SABIC Innov. Pl. US, LLC	329	99	120
PET	-	J125	Mitsui Chemicals Co Ltd.	270	100	150
PET	GF30	Rynite FR530 NC010	DuPont Perf. Polymers	280	110	195
PMMA	-	Plexiglas 8N	Evonik Roehm GmbH	235	70	94
POM	-	Ultraform N2320 003	BASF	200	90	110
PP	-	RJ766MO	Borealis Europe	240	40	111
PP	GF30	Hostacom G3 U01	SF Hoechst	230	50	93
PPS	-	Fortron 6162A7	Celanese	325	147	228
PPS	GF30	Supec G301T	SABIC Innov. Pl. US, LLC	320	150	220
PS	-	Polystyrene 804	TOTAL Petrochemicals	230	50	80

**Table 3:** Thermodynamic properties of the mold materials used for experimental validation.

Mold Property	W300	W600	P20	Copper Alloy
Thermal conductivity (W m^−1^ K^−1^)	26	44.2	29	250
Specific heat capacity (J kg^−1^ K^−1^)	480	473	460	335
Density (kg m^−3^)	7780	7951	7800	8830

**Table 4:** Investigated polymers for the validation with recommended processing temperatures (according to Moldflow material data base of the software version 2016).

Polymer	Filler	Grade Name	Manufacturer	Melt Temp. (°C)	Mold Temp. (°C)	Ejection Temp. (°C)
PVC	-	V-1	Hepworth Building	195	40	68
POM	-	Kepital F10-02	Korea Engineering Plastics Company Ltd.	200	80	135
PP	-	J3021 GR	Prime Polymer Co Ltd.	225	40	100

## Appendix A

**Table A1:** Temperature coefficient (TC), cycle times, and cycle time reduction (CTR) for the investigated polymers for 1 mm part thickness.

Polymer	TC (-)	Cycle Time 1.2343 ESU (s)	Cycle Time Test Alloy (s)	Cycle Time Reduction (CTR) (%)
ABS	4.3	12.3	10.8	11.9
ABS/PC	11.2	18.4	13.9	24.4
PE-HD	3.9	12.3	10.6	13.6
PA 6.6	1.4	6.9	6.8	2.8
PA 6.6 GF30	1.9	8.6	7.6	11.3
PBT	2.4	8.9	8.4	6.0
PBT GF30	6.0	14.9	12.6	14.9
PC	4.7	11.8	10.0	14.6
PC GF30	11.0	18.0	14.0	22.5
PET	3.4	10.4	9.4	9.7
PET GF30	2.0	8.1	7.6	6.1
PMMA	6.9	17.8	14.7	17.6
POM	5.5	24.7	20.8	15.6
PP	2.8	10.6	9.5	10.3
PP GF30	4.2	14.8	12.7	14.0
PPS	2.2	8.0	7.5	6.3
PPS GF30	2.4	7.7	7.4	4.2
PS	6.0	13.0	11.3	13.4

**Table A2:** Temperature coefficient (TC), cycle times, and cycle time reduction (CTR) for the investigated polymers for 3 mm part thickness.

Polymer	TC (-)	Cycle Time 1.2343 ESU (s)	Cycle Time Test Alloy (s)	Cycle Time Reduction (CTR) (%)
ABS	4.3	38.4	35.5	7.6
ABS/PC	11.2	48.8	42.1	13.8
PE-HD	3.9	35.8	32.6	9.0
PA 6.6	1.4	21.4	20.2	5.6
PA 6.6 GF30	1.9	25.8	24.1	6.7
PBT	2.4	29.2	27.2	6.7
PBT GF30	6.0	42.9	37.7	12.2
PC	4.7	34.4	31.5	8.7
PC GF30	11.0	48.5	41.7	13.9
PET	3.4	32.6	30.2	7.1
PET GF30	2.0	21.6	20.1	6.8
PMMA	6.9	55.2	49.1	11.1
POM	5.5	96.1	88.6	7.8
PP	2.8	30.0	27.5	8.4
PP GF30	4.2	46.4	41.8	9.9
PPS	2.2	19.1	17.6	8.0
PPS GF30	2.4	20.8	19.2	7.8
PS	6.0	38.3	35.0	8.6

**Table A3:** Temperature coefficient (TC) and cycle time reductions (CTR) for the validation of the correlation model.

Polymer	TC (-)	CTR Simulated 1 mm Part (%)	CTR Predicted 1 mm Part (%)	CTR Simulated 3 mm Part (%)	CTR Predicted 3 mm Part (%)
PVC	5.5	11.9	14.0	8.6	9.6
POM	2.2	8.0	7.6	7.8	7.0
PP	3.1	12.6	9.4	7.9	7.7

**Table A4:** Deformation results of the open side of the box for the investigated polymers for 1 mm and 3 mm part thickness for both mold materials.

Polymer	Deformation 1 mm Part 1.2343 ESU (%)	Deformation 1 mm Part Test Alloy (%)	Deformation 3 mm Part 1.2343 ESU (%)	Deformation 3 mm Part Test Alloy (%)
ABS	1.61	1.16	0.91	0.77
ABS/PC	0.96	0.72	0.88	0.80
PE-HD	3.77	2.65	2.60	2.06
PA 6.6	2.61	1.89	1.57	1.34
PA 6.6 GF30	2.18	1.83	1.20	1.11
PBT	2.21	1.66	2.50	2.17
PBT GF30	2.23	1.95	1.96	1.82
PC	1.58	1.12	1.13	0.98
PC GF30	1.08	0.90	0.86	0.82
PET	1.66	1.29	1.18	1.06
PET GF30	1.10	0.91	0.84	0.79
PMMA	1.06	0.82	0.78	0.69
POM	1.44	1.05	2.33	2.18
PP	2.92	2.23	1.78	1.48
PP GF30	1.27	1.04	0.81	0.72
PPS	0.73	0.62	0.47	0.44
PPS GF30	0.68	0.55	0.99	0.98
PS	1.3	0.97	0.79	0.68
